# A Novel Computed Tomography-Based Imaging Approach for Etiology Evaluation in Patients With Acute Coronary Syndrome and Non-obstructive Coronary Angiography

**DOI:** 10.3389/fcvm.2021.735118

**Published:** 2021-08-24

**Authors:** Runjianya Ling, Lihua Yu, Zhigang Lu, Yuehua Li, Jiayin Zhang

**Affiliations:** ^1^Institute of Diagnostic and Interventional Radiology, Shanghai Jiao Tong University Affiliated Sixth People's Hospital, Shanghai, China; ^2^Department of Cardiology, Shanghai Jiao Tong University Affiliated Sixth People's Hospital, Shanghai, China; ^3^Department of Radiology, Shanghai General Hospital, Shanghai Jiao Tong University School of Medicine, Shanghai, China

**Keywords:** acute coronary syndrome, coronary, computed tomography angiography, myocardial perfusion imaging, late iodine enhancement

## Abstract

**Objective:** This study sought to investigate the diagnostic value of dynamic CT myocardial perfusion imaging (CT-MPI) combined with coronary CT angiography (CCTA) in acute coronary syndrome (ACS) patients without obstructive coronary angiography.

**Methods:** Consecutive ACS patients with normal or non-obstructive coronary angiography findings who had cardiac magnetic resonance (CMR) contraindications or inability to cooperate with CMR examinations were prospectively enrolled and referred for dynamic CT-MPI + CCTA + late iodine enhancement (LIE). ACS etiology was determined according to combined assessment of coronary vasculature by CCTA, quantified myocardial blood flow (MBF) and presence of LIE.

**Results:** Twenty two patients were included in the final analysis. CCTA revealed two cases of side branch occlusion and one case of intramural hematoma which were overlooked by invasive angiography. High risk plaques were observed in 6 (27.3%) patients whereas myocardial ischemia was presented in 19 (86.4%) patients with varied extent and severity. LIE was positive in 13 (59.1%) patients and microvascular obstruction was presented in three cases with side branch occlusion or spontaneous intramural hematoma. The specific etiology was identified in 20 (90.9%) patients, of which the most common cause was cardiomyopathies (41%), followed by microvascular dysfunction (14%) and plaque disruption (14%).

**Conclusion:** Dynamic CT-MPI + CCTA was able to reveal the potential etiologies in majority of patients with ACS and non-obstructive coronary angiography. It may be a useful alternative to CMR for accurate etiology evaluation.

## Introduction

Acute coronary syndrome (ACS) is one of the leading causes of morbidity and mortality worldwide (1). The concept of ACS usually includes ST-elevation myocardial infarction (STEMI), non-ST-elevation myocardial infarction (NSTEMI) and unstable angina (2). Although the major etiology of ACS lies in acute vessel obstruction secondary to plaque rupture, non-obstructive invasive coronary angiography (ICA) can also be seen in approximately 6% of acute myocardial infarction (AMI) patients (3, 4).

High-sensitivity cardiac troponin I (hs-cTnI) is the most sensitive biomarker for denoting the presence of ACS (5). However, elevation of hs-cTnI can be observed in different clinical scenarios other than AMI caused by coronary artery obstruction. Myocardial infarction with non-obstructive coronary arteries (MINOCA), myocarditis, Takotsubo syndrome and cardiomyopathies may also contribute to acute chest pain, elevated level of hs-cTnI, and similar electrocardiogram change (6).

The management of patients with ACS and non-obstructive coronary arteries depends on the evaluation of underlying etiology, in which cardiac magnetic resonance (CMR) plays an important role for precise diagnosis (7, 8). However, CMR has contraindications, such as claustrophobia and pacemaker implantation. The long acquisition duration and need for strict breath holding further impairs the feasibility of CMR in ACS patients, in whom the image quality is usually suboptimal. Besides, despite of the superiority of myocardial tissue characterization, CMR is unable to visualize the coronary vessel wall anatomy and plaque features which might also provide valuable information for ACS etiology (9, 10).

Dynamic CT myocardial perfusion imaging (CT-MPI) combined with coronary CT angiography (CCTA) has been introduced as a fast one-stop approach for functional and anatomical imaging of coronary artery disease (CAD) (11, 12). Its diagnostic performance for hemodynamic assessment has been validated against invasive fractional flow reserve (FFR) according to previous studies (13, 14). In addition, the clinical application of dynamic CT-MPI + CCTA in AMI patients has also been investigated with reference to CMR (15, 16). Those preliminary studies show promising results in terms of detection of myocardial infarction and microvascular obstruction (MVO) by this CT-based method. Therefore, we hypothesized that dynamic CT-MPI + CCTA might be helpful for etiology evaluation in ACS patients with patent coronary arteries. The aim of the current study was to investigate the diagnostic value of dynamic CT-MPI + CCTA in ACS patients without obstructive coronaries.

## Materials and Methods

### Patient Population

The hospital ethic committee approved this prospective study and all patients gave informed consents. Between March 1st, 2019 and December 31th, 2020, consecutive ACS patients with normal or non-obstructive coronary angiography findings were screened and those who had CMR contraindications or inability to cooperate with CMR examinations were prospectively enrolled. The exclusion criteria were: (1) patients with previous history of coronary revascularization; (2) patients with previous history of myocardial infarction; (3) image quality of dynamic CT-MPI or CCTA was significantly impaired.

The ACS was diagnosed if: (1) patients had acute onset of chest pain; and (2) elevated level of hs-cTnI (>99th percentile of the upper reference level); and (3) excluded other extra-cardiac causes of elevated troponin. Normal or non-obstructive coronary angiography was defined as that invasive coronary angiography (ICA) revealed absence of ≥ 50% stenosis of any epicardial vessel. Subsequently, all patients underwent dynamic CT-MPI + CCTA within 7-days interval after ICA.

### Dynamic CT-MPI + CCTA Acquisition

A third-generation dual source CT (SOMATOM Force, Siemens Healthineers) was used for image acquisition. An integrated protocol, which incorporated calcium score, dynamic CT-MPI, CCTA and late iodine enhancement (LIE), was employed (online Supplement Figure 1). In brief, coronary Agatston calcium score (CACS) was firstly performed to calculate the calcification burden of each epicardial vessels. Intravenous infusion of adenosine triphosphate (ATP) at 160 μg/kg/min was then administrated for 3 min before the triggering of dynamic CT-MPI acquisition. Dynamic CT-MPI was acquired using a shuttle mode technique and started 4 s after the begin of contrast injection. Dynamic acquisition was set at the end-systolic phase (triggered at 250 ms after the R wave in all patients) and scans were launched every second or third heart cycle according to patients' heart rate. CARE kV and CARE dose 4D was used to reduce radiation dose. The reference tube voltage and effective current was 80 kVp and 300 mAs respectively.

Nitroglycerin was given sublingually in all subjects 5 min after dynamic CT-MPI. Prospective ECG-triggered sequential acquisition was performed in all participants for CCTA. 5~7 min after CCTA (17, 18), LIE was acquired using targeted spatial frequency filtration averaging technique (17). The detailed parameters of contrast medium injection, dynamic CT-MPI, CCTA and LIE acquisition were given in online Appendix.

### Image Analysis of CCTA

All CCTA was reconstructed with a smooth kernel (Bv 40) and third generation iterative reconstruction technique (strength 3, ADMIRE, Siemens Healthineers, Germany). Dataset with best image quality from all available phases were transferred to a commercially available workstation (SyngoVia VB 2.0, Siemens Healthineers, Germany) for further analysis. Conventional qualitative and quantitative plaque parameters were evaluated for all lesions on major epicardial arteries (diameter ≥ 2 mm) using a dedicated plaque analysis software (Coronary Plaque Analysis, version 4.3, Siemens Healthineers, Germany). The following indices were measured and recorded: (1) Diameter stenosis (DS); (2) remodeling index and the presence of positive remodeling (PR) (19); (3) low-attenuation plaque (LAP) (20); (4) spotty calcification (SC) (20); (5) Napkin-ring sign (NRS) as defined by previous study (19). The detailed definitions of the above parameters were given in online Appendix. Lesions with at least two HRP features (PR, LAP, SC and NRS) were deemed high-risk plaques (21). The stenosis severity of individuals was evaluated according to Coronary Artery Disease–Reporting and Data System (CAD-RADS) (21).

All CCTA data was independently analyzed by two cardiovascular radiologists (with 12-year and 4-year experience of cardiac imaging) and any disagreement was resolved by consensus.

### Image Analysis of CT-MPI and LIE

Dataset of dynamic CT-MPI was reconstructed with a dedicated kernel (Qr36) for reduction of iodine beam-hardening artifacts. A commercially available CT-MPI software package (Myocardial perfusion analysis, VPCT body, Siemens Healthineers, Forchheim, Germany) was used for further analysis. Motion correction was manually applied if breathing-related mis-registration of the left ventricle was present. The quantification of myocardial blood flow (MBF) was performed using a hybrid deconvolution model, as previously reported (22).

For measurement of absolute MBF, region of interest (ROI) was manually placed on short axis view on a segment base according to the 17-segment model with exclusion of apical segment (23). The segment-based MBF was manually measured with ROI covering the whole myocardial segment (with exclusion of endocardial and epicardial interface). According to the previous studies, MBF < 100 mL/min/100 mL was considered the presence of myocardial ischemia (13, 14).

The dataset of LIE was reconstructed with a dedicated kernel (Qr36) for reduction of iodine beam-hardening artifacts. The four image stacks were averaged to one final image stack using non-rigid registration processed with a volume software (Syngo CT Dynamic Angio, Siemens Healthineers, Germany) in order to reduce image noise. Further multiplanar reformation reconstruction using this fused image stack was made to acquire short axis view of left ventricle, with image thickness and interval of 3 mm. The presence of LIE was defined as focal enhancement with left ventricle myocardium by visual analysis. The LIE patterns were classified into subendocardial, subepicardial, intramural, and diffuse according to the involved areas. Microvascular obstruction (MVO) was defined as the non-enhancing area surrounded by myocardium with LIE (24).

All CT-MPI data was independently analyzed by two cardiovascular radiologists (with 12-year and 4-year experience of cardiac imaging) and any disagreement was resolved by consensus.

### Statistical Analysis

A commercially available software (SPSS Statistics 25, IBM Corp., Armonk, New York) was used for statistical analysis. One-sample Shapiro-Wilk test was used to check the assumption of normal distribution. One-sample *t*-test was used for normally distributed data while Mann-Whitney U test was used for non-normally distributed data. Data for continuous variables were presented as mean ± SD, whereas those with non-normal distribution were presented as median and quartiles. Categorical variables are presented as frequencies and percentages.

## Results

### Patient Characteristics

**Figure 1:**
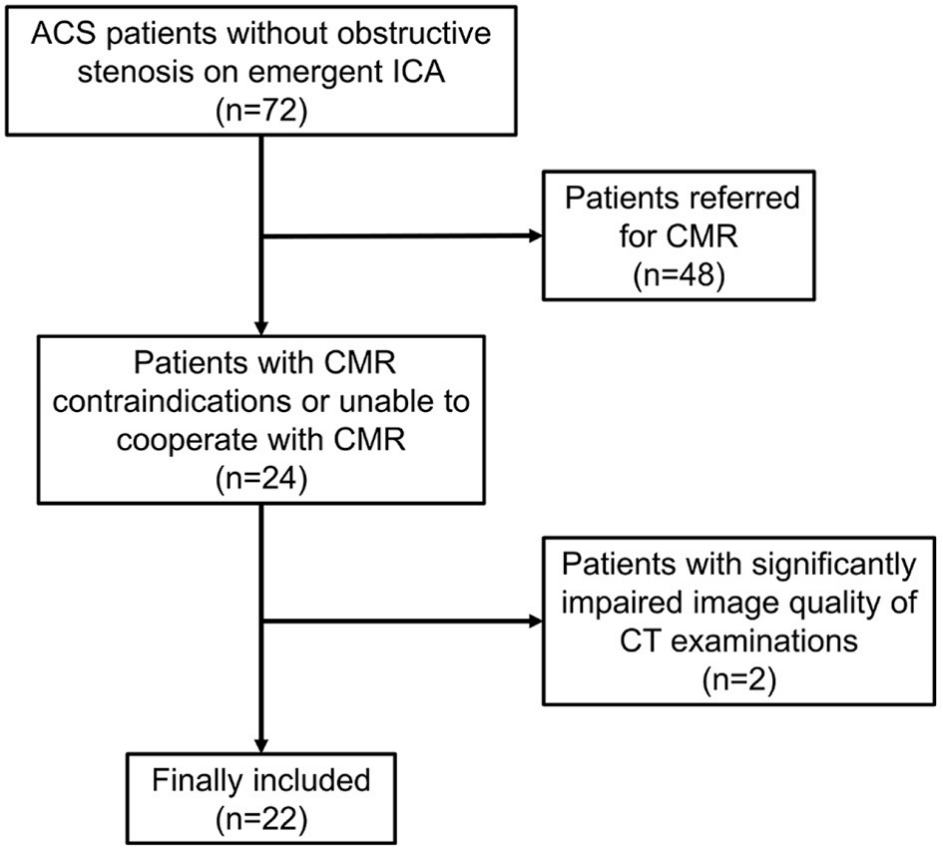
Flow chart of patient inclusion and exclusion. ACS, acute coronary syndrome; CMR, cardiac magnetic resonance; CT, computed tomography; ICA, invasive coronary angiography.

Between March 1st, 2019 and December 31th, 2020, 72 consecutive ACS patients were diagnosed as normal or non-obstructive stenosis on emergent ICA. Among them, 24 patients with CMR contraindications or unable to cooperate with CMR examinations were referred for dynamic CT-MPI and CCTA. Two patients were further excluded due to significantly impaired image quality of CT examinations caused by severe motion artifact (Figure 1). Finally, 22 patients (mean age, 59.50 ± 13.28 years) were included in the current study, including 13 males (mean age, 62.46 ± 11.37 years) and 9 females (mean age, 55.22 ± 15.30 years). The mean radiation dose for dynamic CT-MPI + CCTA (including LIE) was 6.48 ± 2.60 mSv, using 0.014 as the conversion factor. Contrast medium induced nephropathy was not reported in the current study. Detailed demographic data were given in Table 1.

**Table 1:** Clinical characteristics.

**Characteristics**	**Overall**
**Patients (** ***n*** **)**	22 (100 %)
**Males (** ***n*** **)**	13 (59.1 %)
**Age (year)**	59.50 ± 13.28
**Risk factors (** ***n*** **)**	
Current smoker	9 (40.9 %)
Hypertension	17 (77.3 %)
Diabetes mellitus	9 (40.9 %)
Dyslipidemia	3 (15.0 %)
**CT Radiation dose (mSv)**	6.48 ± 2.60
**CKMB elevation (** ***n*** **)**	8 (38.1 %)
**CT contrast medium usage (ml)**	94.73 ± 3.98
**ECG change (** ***n*** **)**
ST-segment elevation	6 (28.6 %)
ST-segment depression	8 (38.1 %)
Non ST-segment change	7 (33.3 %)
**hs-cTnI (ug/L)**	
Onset value	1.07 ± 1.75
Highest value	5.87 ± 9.23

*Values are mean ± SD, or n (%)*.

*CKMB, creatine kinase isoenzyme-MB; ECG, electrocardiogram; hs-cTnI, high-sensitivity cardiac troponin I (normal range 0.00–0.03 ug/L)*.

### Imaging Findings of CCTA

Among ACS patients with normal or non-obstructive coronary angiography, CCTA findings were in line with ICA results in 86.4% (19/22) subjects. The mismatch between CCTA and ICA was due to the overlook of side branch occlusion in two cases and side branch spontaneous intramural hematoma in one case by ICA (Table 2).

**Table 2:** Imaging findings of dynamic CT-MPI + CCTA.

	**Overall**	**Cardiomyopathies**	**Acute myocarditis**	**Ischemic**					**Unclassified**
				**Vascular occlusion**	**Spontaneous intramural hematoma**	**CMD**	**Plaque disruption**	**MB**	
**Patients (** ***n*** **)**	22 (100 %)	9 (40.9 %)	1 (4.5 %)	2 (9.1 %)	1 (4.5 %)	3 (13.6 %)	3 (13.6 %)	1 (4.5 %)	2 (9.1 %)
**CT-MPI**									
Myocardial ischemia (n)	19 (86.4 %)	9 (100 %)	0 (0 %)	2 (100 %)	1 (100 %)	3 (100 %)	3 (100 %)	1 (100 %)	0 (0 %)
Ischemic segments (n)	4.00 (2.75 - 6.00)	4.00 (2.50 - 10.50)	0	3.50 (2.25 - 3.00)	4	5.00 (3.00 - 5.00)	6.00 (4.00 - 6.00)	4	0
Mean MBF of ischemic segment (ml/100ml/min)	70.74 ± 10.02	74.44 ± 6.75	0	47.00 ± 4.24	63	74.17 ± 2.93	75.33 ± 2.31	72	0
**LIE (n)**									
Enhancement	13 (59.1 %)	5 (55.6 %)	1 (100 %)	2 (100 %)	1 (100 %)	0 (0 %)	3 (100 %)	1 (100 %)	0 (0 %)
Within vessel territory	7 (31.8 %)	0 (0 %)	0 (0 %)	2 (100 %)	1 (100 %)	0 (0 %)	3 (100 %)	1 (100 %)	0 (0 %)
MVO	3 (13.6 %)	0 (0 %)	0 (0 %)	2 (100 %)	1 (100 %)	0 (0 %)	0 (0 %)	0 (0 %)	0 (0 %)
**Type (** ***n*** **)**									
Subendocardial	1 (4.5 %)	0 (0 %)	0 (0 %)	0 (0 %)	0 (0 %)	0 (0 %)	1 (33.3 %)	0 (0 %)	0 (0 %)
Intramural	1 (4.5 %)	1 (11.1 %)	0 (0 %)	0 (0 %)	0 (0 %)	0 (0 %)	0 (0 %)	0 (0 %)	0 (0 %)
Subepicardial	1 (4.5 %)	0 (0 %)	1 (100 %)	0 (0 %)	0 (0 %)	0 (0 %)	0 (0 %)	0 (0 %)	0 (0 %)
Diffuse	10 (45.5 %)	4 (44.4 %)	0 (0 %)	2 (100 %)	1 (100 %)	0 (0 %)	2 (66.7 %)	1 (100 %)	0 (0 %)
**CCTA (** ***n*** **)**									
**CAD-RADS category**									
CAD-RADS 0	2 (9.1 %)	1 (11.1 %)	1 (100 %)	0 (0 %)	0 (0 %)	0 (0 %)	0 (0 %)	0 (0 %)	0 (0 %)
CAD-RADS 1	3 (13.6 %)	1 (11.1 %)	0 (0 %)	0 (0 %)	0 (0 %)	1 (33.3 %)	0 (0 %)	0 (0 %)	1 (50 %)
CAD-RADS 2	14 (63.6 %)	7 (77.7 %)	0 (0 %)	0 (0 %)	0 (0 %)	2 (66.7 %)	3 (100 %)	1 (100 %)	1 (50 %)
CAD-RADS 3	0 (0 %)	0 (0 %)	0 (0 %)	0 (0 %)	0 (0 %)	0 (0 %)	0 (0 %)	0 (0 %)	0 (0 %)
CAD-RADS 4	1 (4.5 %)	0 (0 %)	0 (0 %)	0 (0 %)	1 (100 %)	0 (0 %)	0 (0 %)	0 (0 %)	0 (0 %)
CAD-RADS 5	2 (9.1 %)	0 (0 %)	0 (0 %)	2 (100 %)	0 (0 %)	0 (0 %)	0 (0 %)	0 (0 %)	0 (0 %)
**Plaque features (** ***n*** **)**									
LAP	3 (13.6 %)	0 (0 %)	0 (0 %)	2 (100 %)	0 (0 %)	0 (0 %)	1 (33.3 %)	0 (0 %)	0 (0 %)
PR	11 (50.0 %)	2 (22.2 %)	0 (0 %)	2 (100 %)	0 (0 %)	3 (100 %)	3 (100 %)	0 (0 %)	1 (50 %)
SC	7 (31.8 %)	2 (22.2 %)	0 (0 %)	2 (100 %)	0 (0 %)	0 (0 %)	2 (66.7 %)	1 (100 %)	0 (0 %)
NRS	2 (9.1 %)	1 (11.1 %)	0 (0 %)	0 (0 %)	0 (0 %)	0 (0 %)	1 (33.3 %)	0 (0 %)	0 (0 %)
High-risk plaques	6 (27.3 %)	1 (11.1 %)	0 (0 %)	2 (100 %)	0 (0 %)	0 (0 %)	3 (100 %)	0 (0 %)	0 (0 %)

*Values are mean ± SD, n (%), or median (interquartile range)*.

*CCTA, coronary CT angiography; CMD, coronary microvascular dysfunction; LAP, low-attenuation plaque; LIE, late iodine enhancement; MB, myocardial bridge; MBF, myocardial blood flow; MPI, myocardial perfusion imaging; MVO, microvascular obstruction; NRS, napkin-ring sign; PR, positive remodeling; SC, spotty calcification*.

Overall, CCTA revealed normal coronary arteries (CAD-RADS 0) in 2 (9.1 %) patients, non-obstructive stenosis (CAD-RADS 1-2) in 17 (77.3 %) patients, and obstructive stenosis (CAD-RADS 3-5) in 3 (13.6 %) patients (Table 2). In terms of HRP features, PR and SC were commonly presented in the current population whereas HRP was observed in 6 (27.3%) patients (Table 2).

### Imaging Findings of Dynamic CT-MPI

Myocardial ischemia was presented in 19 (86.4 %) patients with varied extent and severity. Lowest MBF was observed in the cases with overlooked side branch occlusion, followed by the case with spontaneous intramural hematoma (Table 2).

In addition, LIE was positive in 13 (59.1 %) patients and diffuse LIE was the most common pattern (Table 2). LIE area complied with vessel-related territory in seven cases and randomly distributed in six cases. MVO was presented in three cases with side branch occlusion or spontaneous intramural hematoma.

### Etiology of ACS According to Dynamic CT-MPI + CCTA

The underlying etiologies of ACS were subsequently classified into ischemic, cardiomyopathies, acute myocarditis and unclassified, according to the imaging findings of dynamic CT-MPI + CCTA (Table 2).

**Figure 2:**
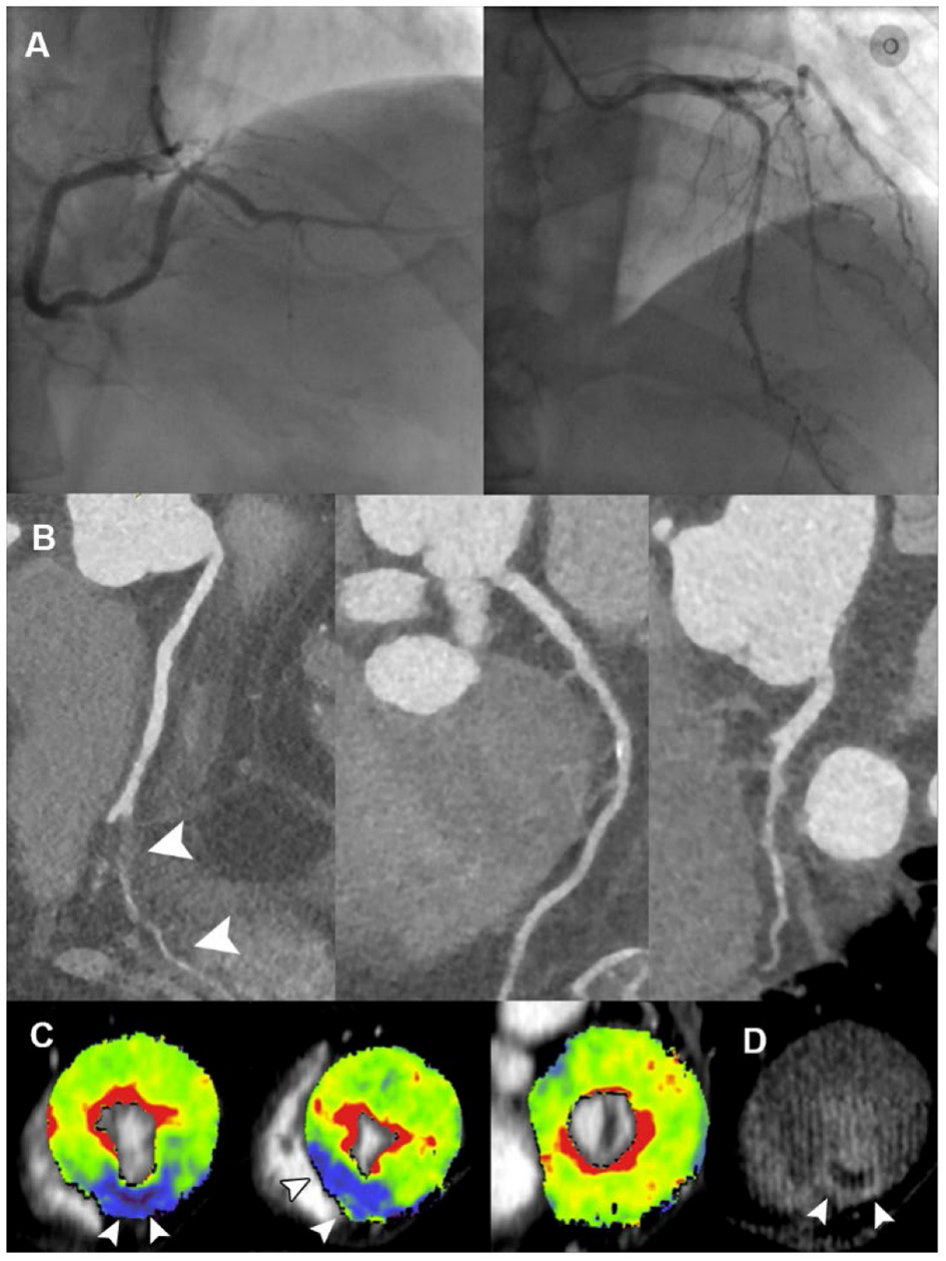
Representative case of vessel occlusion with MVO in a 66-year-old male with ACS and non-obstructive ICA. **(A)** ICA revealed mild stenosis at middle LAD and distal RCA, without presence of obstructive lesion. **(B)** CPR images showed occlusion of PDA ostium (white arrowhead). **(C)** Short-axis views of CT-MPI showed significantly reduced myocardial blood flow within PDA territory (white arrowhead). **(D)** LIE image showed the transmural late enhancement with the presence of MVO within PDA territory (white arrowhead). ACS, acute coronary syndrome; CPR, curved planar reformation; CT, computed tomography; ICA, invasive coronary angiography; LAD, left anterior descending; LIE, late iodine enhancement; MPI, myocardial perfusion imaging; MVO, microvascular obstruction; PDA, posterior descending artery; RCA, right coronary artery.

**Figure 3:**
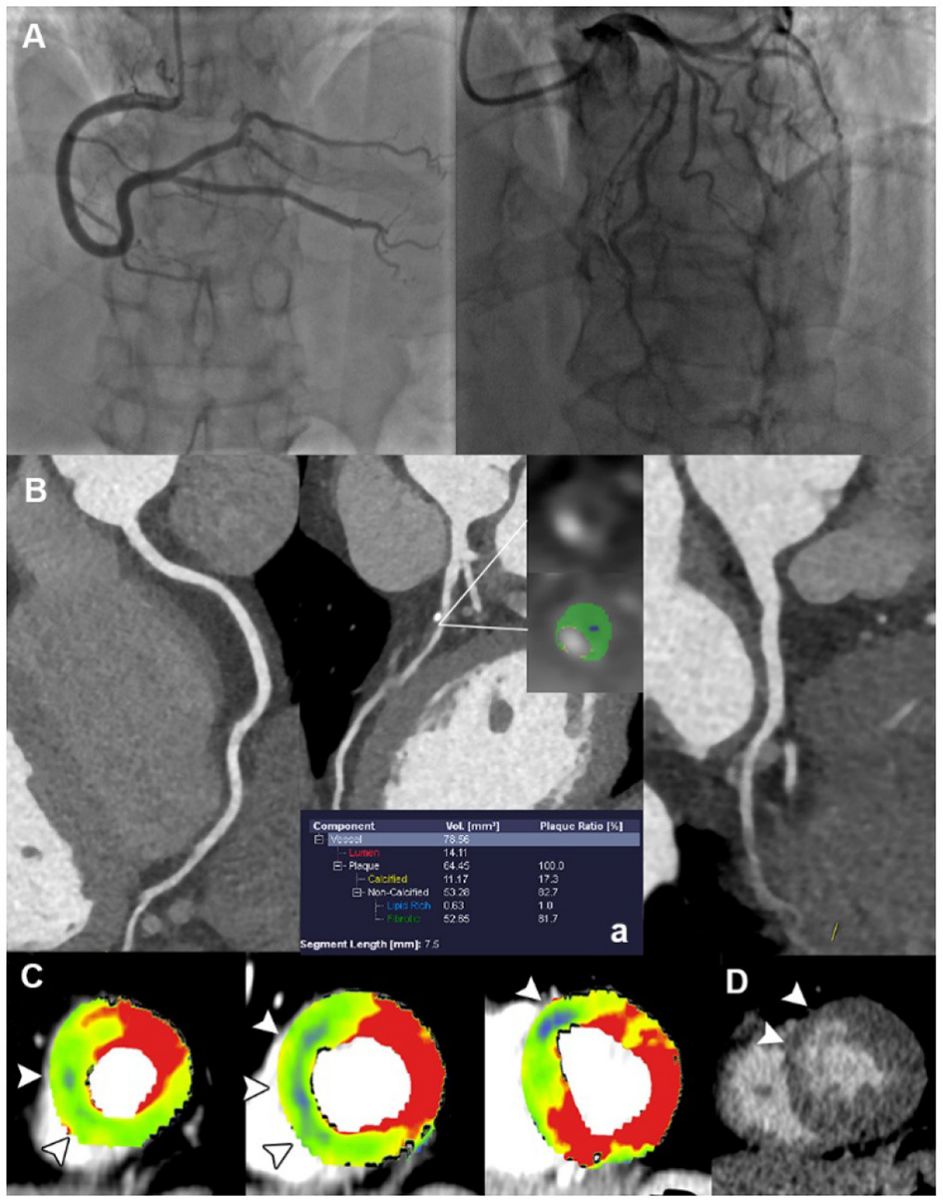
Representative case of plaque disruption in a 61-year-old male with ACS and non-obstructive ICA. **(A)** ICA revealed mild stenosis at proximal LAD, without presence of obstructive lesion. **(B)** CPR images showed mixed plaque at proximal LAD with the presence of LAP, PR, NRS, and SC, indicating a typical HRP. **(C)** Short-axis views of CT-MPI showed significantly reduced myocardial blood flow within LAD territory (white arrowhead). **(D)** LIE image showed the subendocardial late enhancement within LAD territory (white arrowhead). ACS, acute coronary syndrome; CPR, curved planar reformation; CT, computed tomography; ICA, invasive coronary angiography; LAD, left anterior descending; LAP, low-attenuation plaque; LIE, late iodine enhancement; MPI, myocardial perfusion imaging; MVO, microvascular obstruction; NRS, napkin-ring sign; PR, positive remodeling; SC, spotty calcification.

**Figure 4:**
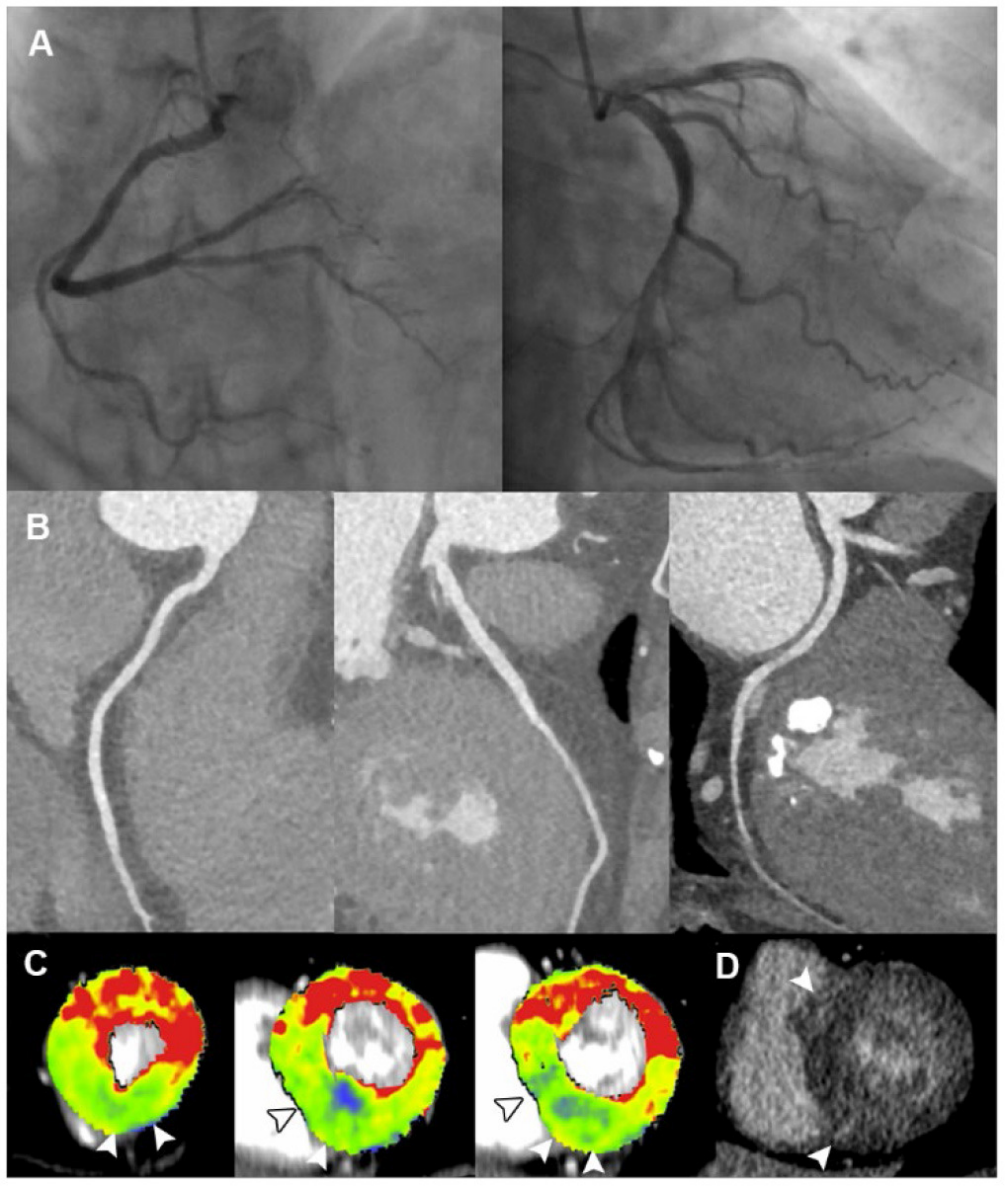
Representative case of cardiomyopathy in a 60-year-old female with ACS and non-obstructive ICA. **(A,B)** ICA and CCTA revealed normal coronary vasculature. **(C)** Short-axis views of CT-MPI showed significantly reduced myocardial blood flow at apical inferior, mid septal, mid inferior, basal septal as well as basal inferior segments, with focal myocardial hypertrophy (white arrowhead). **(D)** LIE image showed the focal intra-mural late enhancement at RV insertion points (white arrowhead). The above findings were consistent with the diagnosis of hypertrophic cardiomyopathy. ACS, acute coronary syndrome; CCTA, coronary computed tomography angiography; CT, computed tomography; ICA, invasive coronary angiography; LAD, left anterior descending; LAP, low-attenuation plaque; LIE, late iodine enhancement; MPI, myocardial perfusion imaging; MVO, microvascular obstruction; RV, right ventricle.

The specific ischemic causes of ACS could be further divided into vessel occlusion (defined as presence of side branch occlusion, reduced MBF and positive LIE within vessel territory, Figure 2), spontaneous intramural hematoma (defined as long segment of continuous filling defect within lumen, reduced MBF and positive LIE within vessel territory), plaque disruption (defined as presence of HRP features, non-obstructive stenosis, reduced MBF and positive LIE within vessel territory) (Figure 3), microvascular dysfunction (defined as reduced MBF with random distribution and absence of LIE), and myocardial bridging (defined as reduced MBF and positive LIE within myocardial bridging territory). Other causes of ACS included cardiomyopathies (absence of obstructive stenosis, presence of hypertrophic or dilated left ventricle, with or without myocardial ischemia/LIE) (Figure 4) and acute myocarditis (absence of epicardial stenosis, normal myocardial perfusion, and presence of typical subepicardial LIE).

**Figure 5:**
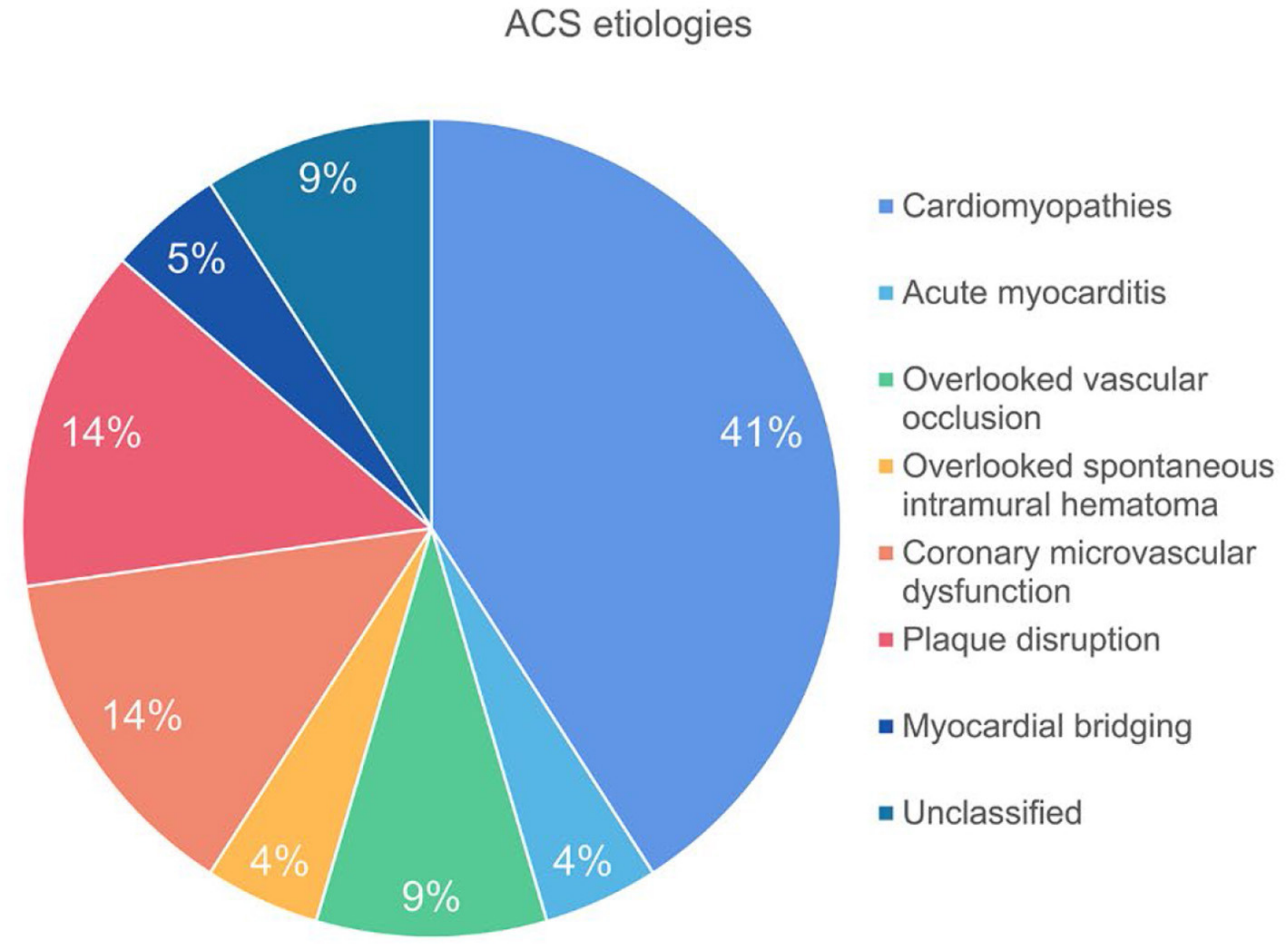
Pie chart of ACS etiologies.

Finally, there were two cases diagnosed as “unclassified” due to the absence of imaging evidence of myocardial ischemia or injury. Thus, the specific etiology was identified in 20 (90.9 %) patients by dynamic CT-MPI + CCTA (Figure 5).

## Discussion

The major finding of the present study showed that dynamic CT-MPI + CCTA was able to uncover the underlying etiologies in majority of patients with ACS and non-obstructive coronary angiography.

Normal or non-obstructive ICA is not an uncommon finding in AMI patients (3, 4) and the specific etiologies in this cohort consist of not only coronary causes (epicardial causes, including plaque disruption and coronary artery dissection, microvascular causes, such as microvascular dysfunction and coronary artery spasm), but also Takotsubo syndrome, acute myocarditis, and cardiomyopathies (7, 25). Identification of the underlying etiology in those subjects is of paramount importance because the clinical management and prognosis strongly depend on the precise diagnosis. According to a scientific statement from the American Heart Association regarding diagnosis and management of MINOCA, CMR is recommended as a key investigation in the diagnostic work-up because it can exclude myocarditis, takotsubo syndrome, and cardiomyopathies, as well as provide imaging confirmation of AMI (6). According one recent study, CMR combined with optical coherence tomography (OCT) was able to identify potential mechanisms in 84.5% of women with a diagnosis of MINOCA (26). However, CMR and OCT are still not widely available imaging approaches and ACS patients might not be able to cooperate with the long acquisition time of CMR. Thus, developing an alternative imaging modality with the ability of assessing myocardial injury and potential etiology would be clinically beneficial.

The current study for the first time investigated the clinical value of dynamic CT-MPI + CCTA in the diagnostic workflow of ACS without obstructive coronary angiography. Thanks to the technical advantages of this “one-stop shop” imaging modality, this CT-based approach allows simultaneous assessment of coronary vasculature, myocardial perfusion, and myocardial injury as well. As one core part of the CT protocol, dynamic CT-MPI is able to accurately quantify MBF and diagnose myocardial ischemia (13, 14). LIE has also been proven to represent a useful alternative to late gadolinium enhancement to detect myocardial scar (27). Moreover, CCTA provides comprehensive evaluation of coronary anatomy, including overlooked side branch occlusion, intramural hematoma, and HRP features of suspected culprit lesions. Although it is not a direct imaging method for diagnosis of plaque disruption, CCTA has been validated for identification of thin-cap fibroatheroma with reference to intravascular ultrasound (28). It complements dynamic CT-MPI for exploration of potential epicardial etiologies when myocardial injury is presented. With the help of the above comprehensive assessment, dynamic CT-MPI + CCTA was able to identify the specific etiology of ACS without obstructive ICA in 90.9 % patients in the current cohort, which was similar to one previous CMR + OCT study (26).

In light of the above findings, the clinical role of dynamic CT-MPI + CCTA lies in the following aspects for ACS evaluation. First, this CT-based approach provides a useful alternative to CMR for etiology assessment in ACS patients without obstructive coronary angiography. Unlike CMR, which requires longer acquisition time and has more contraindications, dynamic CT-MPI + CCTA offers a faster way for functional and anatomical evaluation. Although dynamic CT-MPI + CCTA is currently unable to visualize myocardial edema, it is still a valuable complement in cases with CMR contraindications for etiology investigation. ACS patients without obstructive ICA may benefit from this CT-based method owing to specific etiology identification and precise treatment accordingly. Moreover, thanks to the technical development, the radiation exposure of CT-MPI + CCTA decreases significantly, from 13.1 to 6.3 mSV (13, 29). In the current study using third-generation dual source CT, the overall radiation dose of whole CT protocol (including LIE) was 6.48 mSv, which was reasonable and acceptable in clinical practice.

Despite the above promising results, the current study has several limitations. First, CMR and OCT validation were not available in the present study to confirm the diagnosis by dynamic CT-MPI + CCTA. Thus, the diagnostic accuracy of CT-based etiology evaluation could not be assessed. In addition, the overall sample size was small in this preliminary study with limited case number of different etiologies, which made inter-group analysis unavailable. The prognostic value of CT imaging was also unclear. Future studies with larger sample size are warranted to investigate the clinical impact of CT-based approach on guiding proper management and prognosis improvement. Finally, due to technical limitation, it is of note that dynamic CT-MPI + CCTA was unable to evaluate several pathological features, such as myocardial edema, systolic function of left ventricle, coronary emboli and coronary spasm. Therefore, some MINOCA etiologies, such as Takostubo Syndrome, coronary thrombus and coronary spasm, cannot be assessed using this approach. Other imaging methods should be employed when these causes are suspected.

In conclusion, dynamic CT-MPI + CCTA was able to reveal the potential etiologies in majority of patients with ACS and non-obstructive coronary angiography. It may be an useful alternative to CMR for accurate etiology evaluation.

## Data Availability Statement

The raw data supporting the conclusions of this article will be made available by the authors, without undue reservation.

## Ethics Statement

The studies involving human participants were reviewed and approved by Shanghai Jiaotong University Affiliated Sixth People's Hospital Ethics Committee. The patients/participants provided their written informed consent to participate in this study.

## Author Contributions

RL contributed to image analysis, statistical analysis, and manuscript drafting. LY contributed to image analysis and statistical analysis. ZL contributed to patient enrollment and image analysis. YL contributed to literature research and manuscript drafting. JZ contributed to study conception and design, image analysis, literature research, and manuscript drafting.

## Conflict of Interest

The authors declare that the research was conducted in the absence of any commercial or financial relationships that could be construed as a potential conflict of interest.

## Publisher's Note

All claims expressed in this article are solely those of the authors and do not necessarily represent those of their affiliated organizations, or those of the publisher, the editors and the reviewers. Any product that may be evaluated in this article, or claim that may be made by its manufacturer, is not guaranteed or endorsed by the publisher.
