# Supplementary material for: A Novel Computed Tomography-Based Imaging Approach for Etiology Evaluation in Patients With Acute Coronary Syndrome and Non-obstructive Coronary Angiography
Source: Front Cardiovasc Med. 2021 Aug 24;8:735118. doi: 10.3389/fcvm.2021.735118 (PMC8421729; doi:10.3389/fcvm.2021.735118)
Supplement: Supplementary file 1 [file Data_Sheet_1.docx]

Online appendix

1. Dynamic CT-MPI + coronary CT angiography protocol

Calcium score was firstly performed to calculate the calcification burden of each pericardial vessels. The scan range of dynamic CT-MPI was determined based on the calcium score images to cover the whole left ventricle as well as all coronary arteries. Adenosine triphosphate was intra-venously infused over 3 min at 160 µg/kg/min before triggering the MPI acquisition. A fixed volume of contrast media (50ml, Ultravist, 370 mg iodine/ml, Bayer) was given in a bolus injection at the rate of 6 ml/s in all participants, followed by a 40 ml saline flush by using dual-barrel power injector (Tyco, Cincinnati, US). Dynamic CT-MPI acquisition was started 4 s after the begin of contrast injection. The end-systolic phase (triggered at 250 ms after the R wave in all participants) was set for the dynamic acquisition by using a shuttle mode technique with a coverage of 10.5 cm for complete imaging of the whole left ventricle. Scans were launched every second or third heart cycle according to participants’ heart rate, resulting in a series of 10 to 15 phases acquired over a fixed period of 32 s. The acquisition parameters of dynamic CT-MPI is listed as follow: collimation = 96×0.6 mm, CARE kV was used and the reference tube voltage= 80 kVp, rotation time = 250 ms, CARE dose 4D was used and the effective current= 300 mAs, reconstructed slice thickness = 3 mm and reconstructed slice interval = 2 mm.

Nitroglycerin was given sublingually in all participants 5 minutes after dynamic CT-MPI, prior to the acquisition of coronary CT angiography. Coronary CT angiography was performed by using a bolus tracking technique, with regions of interest placed in the ascending aorta. A bolus of contrast media was injected into antecubital vein at the rate of 4-5 ml/s, followed by a 40 ml saline flush by using dual-barrel power injector. The amount of the contrast media was determined according to the patient's body weight (patients with body mass index < 18 injected with 40 mL contrast media at 4 ml/s, patients with body mass index between 18 and 24 injected with 50 mL contrast media at 4.5 ml/s, patients with body mass index > 24 injected with 60 mL contrast media at 5 ml/s). Prospective ECG-triggered sequential acquisition was performed in all participants for coronary CT angiography, with the acquisition window covering from 35% to 75% of R-R interval, with collimation = 96×0.6 mm, reconstructed slice thickness = 0.75 mm, reconstructed slice interval = 0.5mm, rotation time = 250 ms and application of automated tube voltage and current modulation (CAREKv, CAREDose 4D, Siemens Healthineers). The reference tube current was set as 320 mAs and the reference tube voltage was set as 100 kVp.

Delayed iodine enhancement scan was performed 5 to 7 minutes after coronary CT angiography, using targeted spatial frequency filtration averaging technique. In brief, a shuttle mode scan with a coverage of 10.5 cm was employed. The acquisition parameters were set as same as those of dynamic CT-MPI, except for shorter duration (set for including 4 phases).

2. Image analysis of CCTA

CCTA data was reconstructed with smooth kernel (Bv 40) and third generation iterative reconstruction (IR) technique (strength 3, ADMIRE, Siemens). A dedicated research software (Coronary Plaque Analysis, version 4.3, Siemens Healthineers, Germany) was employed for further analysis of all lesions with stenosis extent ≧ 30% at any epicardial vessel with diameter ≧2mm. The following indices were measured and recorded: 1) Diameter stenosis (DS) was calculated as (reference diameter – minimal lumen diameter) / reference diameter and was measured manually with a digital caliper at the narrowest level of the lesion and the proximal reference on the cross-sectional images; 2) Remodeling index was defined as a maximal lesion vessel diameter divided by proximal reference vessel diameter (at the site where no plaque component can be detected), with positive remodeling (PR) defined as a remodeling index ≥ 1.1; 3) Low-attenuation plaque (LAP) was defined as any voxel < 30 HU within a coronary plaque; 4) Spotty calcification (SC) was defined by an intra-lesion calcific plaque < 3 mm in length that comprised < 90 degrees of the lesion circumference; 5) Napkin-ring sign (NRS) was characterized by a plaque core with low attenuation areas on CT surrounded by a rim-like area of higher attenuation as previously reported. Lesions with at least two high-risk plaque features (PR, LAP, SC and NRS) were deemed high-risk plaques (HRPs). The coronary stenosis of individuals was evaluated according to Coronary Artery Disease - Reporting and Data System (CAD-RADS).

All the above parameters were independently analyzed by two cardiovascular radiologists (with 12-year and 4-year experience of cardiac imaging) and the mean values of measurement were used for further analysis.
