# Supplementary figures and images for: A Novel Computed Tomography-Based Imaging Approach for Etiology Evaluation in Patients With Acute Coronary Syndrome and Non-obstructive Coronary Angiography
Source: Front Cardiovasc Med. 2021 Aug 24;8:735118. doi: 10.3389/fcvm.2021.735118 (PMC8421729; doi:10.3389/fcvm.2021.735118)

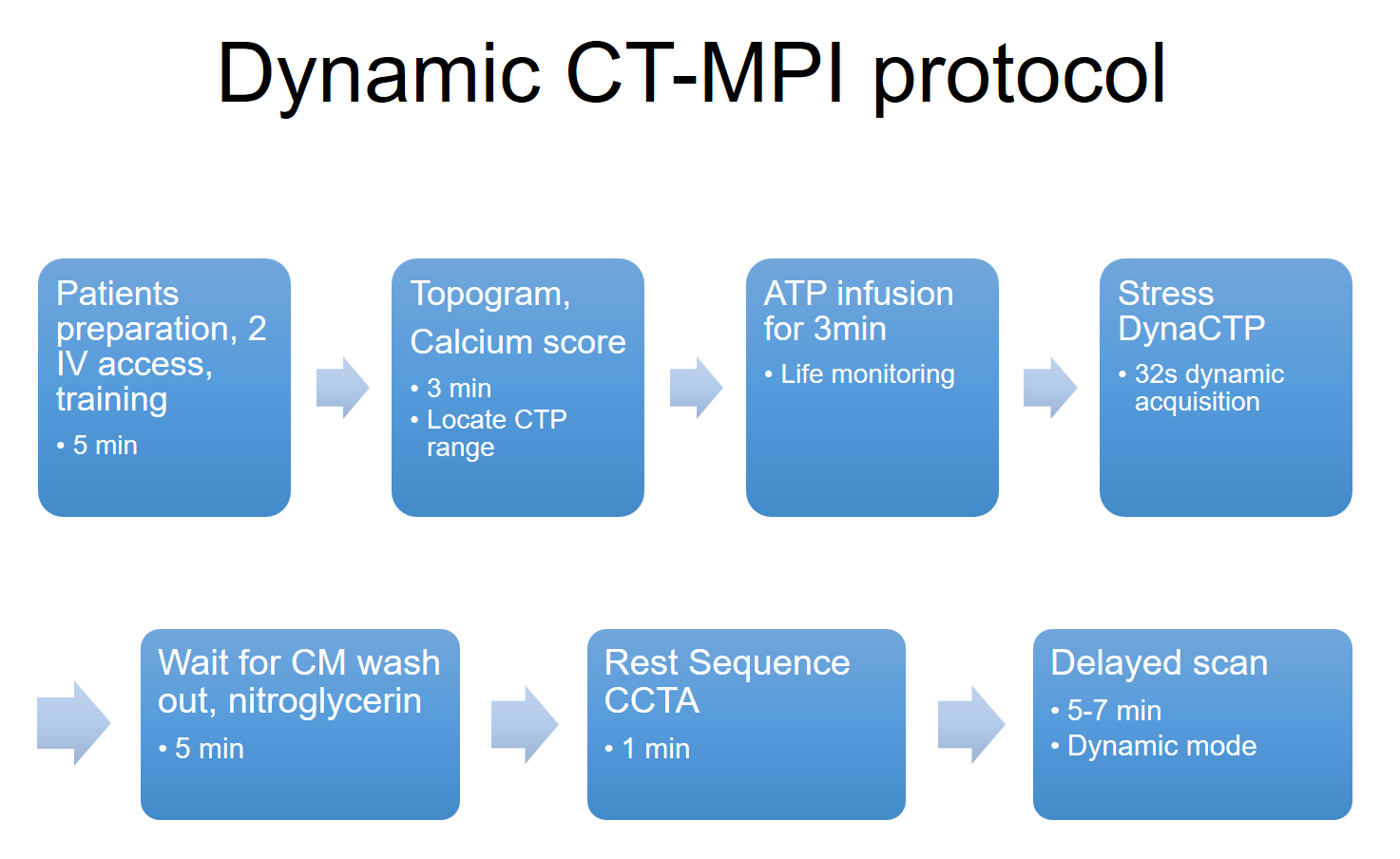

Supplement: Supplementary file 2 [file Image_1.TIFF]
